# Tackling climate change at the city level: insights from Lighthouse Cities' climate mitigation efforts

**DOI:** 10.3389/fpsyg.2023.1308040

**Published:** 2024-02-08

**Authors:** Mehmet Efe Biresselioglu, Zehra Funda Savas, Muhittin Hakan Demir, Cigdem Kentmen-Cin

**Affiliations:** ^1^Sustainable Energy Division, Izmir University of Economics, Izmir, Türkiye; ^2^Department of Logistics Management, Izmir University of Economics, Izmir, Türkiye; ^3^Department of Political Science and International Relations, Izmir University of Economics, Izmir, Türkiye

**Keywords:** climate mitigation, lifestyle change, expert survey, lighthouse cities, climate change

## Abstract

**Introduction:**

The link between lifestyles and Greenhouse Gas (GHG) emissions has prioritized climate mitigation strategies of cities worldwide. As cities have increasingly generated GHG emissions by their industrial and transportation activities, their role in climate mitigation has gained prominence. Cities' climate mitigation policies to reduce the GHG intensity of their residents' daily lives are one of their significant efforts to tackle climate change. Lighthouse Cities (LCs), in particular, have emerged as remarkable actors in promoting lifestyle changes for their residents.

**Methods:**

This study examines climate mitigation strategies of LCs of Climate CAMPAIGNers project, including Baku, Vilnius, Lahti, Izmir, Trujillo, Athens, Linz, Milan, Cape Town, Dublin, and Skopelos, addressing lifestyle changes by conducting an expert survey in 11 LCs involving 89 respondents. The findings of the expert survey are comparatively analyzed across 11 LCs.

**Results:**

The results show that experts form Lighthouse Cities identify increasing awareness and information provision as a significant component of climate mitigation policies. Concerning lifestyle changes, strategies toward energy efficiency and sustainable mobility are highlighted as the primary areas to be prioritized.

**Discussion:**

This study enhances the understanding of cities' capacity to reduce their residents' GHG emissions. The findings can be utilized to identify and tailor policies for supporting the Lighthouse Cities in their climate change mitigation efforts and provide pointers for selecting the lifestyle changes that can be promoted and prioritized in Lighthouse Cities.

## 1 Introduction

The nexus between lifestyles and GHG emissions has been increasingly part of the policy agendas of cities worldwide. As climate change has become a global emergency, individual lifestyle changes have been prioritized in addition to local and national policies. Changing habits, behaviors, and consumption patterns can reduce environmental impact and emissions across various sectors and lifestyles. To reach the goals of a “net-zero future” and the Paris Agreement, the United Nations (UN) has initiated the “ActNow” campaign, which seeks to guarantee individual lifestyle changes toward reducing GHG emissions (United Nations, 2023a).

Cities stand out as essential actors taking climate action to promote lifestyle changes for their residents against the negative consequences of climate change. They are significant because cities are responsible for more than 70% of CO_2_ emissions in the world with their industries and transportation networks (Dasgupta et al., 2022). They also have resources and prosperity to fight climate change in addition to their “pro-activity” and “network-like” strategies (Rosenzweig et al., 2010; Eisenack and Roggero, 2022). Furthermore, their populations, infrastructure, and economies are exposed to several outputs of climate change, such as a rise in “sea level” and intensive “droughts” (Rosenzweig et al., 2010).

Various cities worldwide have noticed the adverse consequences of climate change and have sought to take climate action to contribute to their residents' lifestyle changes. Climate change mitigation is one of the significant actions that cities undertake. Climate change mitigation involves all the strategies to minimize GHG emissions (United Nations, 2023b). Promoting their residents' lifestyle changes is one of city authorities' most prominent climate actions in the face of the global climate crisis (Quam et al., 2017; Sun and Feng, 2023; Zhang and Zheng, 2023).

In this context, Lighthouse City (LC) projects have been increasingly initiated by the European Union (EU) to support cities' ability to “develop and test integrated innovative solutions at district scale” (European Commission, 2016). Several LCs under the Horizon 2020 programs have been chosen for their efforts to pursue energy efficiency strategies and the Sustainable Energy Action Plan (SEAP). Hence, LCs are crucial actors in encouraging their citizens to “engage in climate action” (Climate Campaigners, 2023).

Within this framework, this study aims to examine the policy levers that can influence daily lifestyles in LCs of the Climate CAMPAIGNers project: Baku, Vilnius, Lahti, Izmir, Trujillo, Athens, Linz, Milan, Cape Town, Dublin, and Skopelos. In order to answer the research question “How can climate change mitigation policies that address lifestyle transformation in LCs be operationalised?”, input from the LCs is obtained through the expert survey developed by the academic partners and completed by experts from the relevant LCs.

The manuscript is organized as follows. The next section on methodology discusses the comprehensive state-of-the-art literature review and the expert survey. A comprehensive, state-of-the-art literature review demonstrates how cities' climate mitigation strategies and residents' lifestyle changes are analyzed in earlier research. The third section provides the significant outputs of the expert survey. Then, the discussion section presents a comparative analysis of survey findings across different LCs regarding lifestyle changes and climate mitigation. Lastly, the motivators and barriers to feasible climate actions of LCs are discussed in the Conclusion section.

## 2 Methodology and research design

This study relies on a comprehensive, state-of-the-art literature review and an expert survey to examine the policy levers influencing lifestyle changes and climate mitigation strategies in LCs. Overall, the research framework of this study consists of nine subsequent steps, including (1) Preparation of the template and guidelines for a state-of-the-art literature review, (2) conducting the state-of-the-art literature review, (3) analysis of the results of the literature review, (4) design of the expert survey, (5) fine-tuning, pre-test, and pilot of the expert survey, (6) selecting the sample for expert survey using purposive sampling, (7) conducting the expert survey, (8) analysis of the results of expert survey, and (9) synthesis of the results of the state-of-the-art literature review and expert survey.

This study conducted a comprehensive, state-of-the-art literature review using a predefined template to maintain methodological consistency. The authors designed the template to ensure focused information collection directly relevant to this study's objectives and research question, encompassing parameters investigated, research methodology, and findings. Accordingly, the literature review template was designed in two sections. The first section, state-of-the-art, provides a comprehensive examination of existing studies and their understanding of the terms “climate change,” “climate mitigation,” “Lighthouse Cities,” “climate-harming lifestyles,” “climate-friendly lifestyles,” and “energy behavior.” The second part of the literature review concentrates on the relevance of these studies to the conceptualization of “climate change,” “climate mitigation,” and “lifestyles,” ensuring an in-depth understanding of these concepts.

The findings from the literature review are used to design the expert survey, which aims to collect information from the experts in the Lighthouse Cities regarding their perspectives on past, current, and potential climate mitigation and adaptation strategies. This manuscript utilizes findings from the expert survey regarding the climate change mitigation strategies. The expert survey methodology has been frequently used in the literature to understand the expert attitudes about a particular issue in local and national affairs (Groholt and Higley, 1972; Saiegh, 2009; Kertzer and Renshon, 2022). It is an effective method to analyze the perspectives of individuals with extensive knowledge, authority, or experience about a specific issue. For this survey, respondents were selected through purposeful sampling, targeting experts with extensive experience planning and implementing climate mitigation policies within their respective LCs. This method was utilized to ensure that the selected participants had professional knowledge about climate mitigation strategies, lifestyle changes, and related experience at the local level. The final sample included 89 experts form 11 LCs involving 15 individuals from Baku (Azerbaijan), 12 from Vilnius (Lithuania), 11 from Lahti (Finland), 10 each from Izmir (Türkiye) and Trujillo (Peru), 9 from Athens (Greece), 8 from Linz (Austria), 5 from Milan (Italy), and 3 each from Cape Town (South Africa), Dublin (Ireland), and Skopelos (Greece).

All participants have professional knowledge about climate mitigation strategies and lifestyle changes and related experience at the local level. Their LCs are situated within the countries of Climate CAMPAIGNers project partners and cover a diverse geographical area that includes both southern and northern regions, the European Union (EU) and non-EU members. The participants represent a spectrum of relevant roles, including mid-level and senior municipal officers, faculty members, researchers, advisors, private company representatives, professional chamber members, climate action planners, and NGO members. The largest category involves mid-level officers, with 29 respondents. This category is comprised of officers in “transportation,” “environmental/sustainability,” “construction,” “finance,” and other duties in cities. The second prominent group includes faculty members and researchers, with 21 respondents. These two professions are followed by representatives of private companies (*n* = 14), advisors (*n* = 13), members of professional chambers (*n* = 6), members of climate action planning (*n* = 6), senior officers (*n* = 3), representatives of NGOs (*n* = 2), executives (*n* = 1) and miscellaneous experts who have relevant expertise (*n* = 5).

The survey was designed to capture the in-depth insights and perspectives of experts (Patton, 2002, p. 273). Accordingly, the survey is designed in three parts. The first part involves questions concerning LCs' climate change mitigation strategies, the second part seeks to assess LCs' efforts toward lifestyle changes, and the third part focuses on the climate adaptation strategies, from the perspective of each expert. This manuscript is based on the results from the first two parts of the survey.

Concerning the first part of the survey, the literature review revealed five main themes regarding policy actions for climate mitigation: “changes in lifestyles,” “education and enabling,” “financing and provision,” “information and communication technologies (ICT) and digitalization,” “municipal self-governing,” and “regulation.” To assess whether these themes align with experts' concerns in LCs, the first survey question asked respondents to select the top three climate mitigation policies and tools their LCs prioritize. The subsequent questions in the first part of the survey asked respondents about the primary policy actions implemented in the last 5–10 years and are currently being implemented and/or need to be implemented in the next 5–10 years to deal with climate mitigation in the respective relevant cities.

The second part of the expert survey concerns the LCs' efforts toward lifestyle changes. To this end, the participants were first asked to select the top five lifestyle changes for climate policies that the experts find essential for their LCs. As with the policy action counterparts, the experts were also asked in the second part about the lifestyle choices that last 5–10 years and are currently being discussed and/or need to be discussed in the next 5–10 years to improve the LCs' climate policies. The final set of survey questions aimed to identify experts' perspectives regarding motivators and barriers to lifestyle changes in the context of climate policymaking.

The survey was constructed as a questionnaire, including inquiries on expert information, climate mitigation policy actions, and the LCs' efforts toward lifestyle changes. The survey was distributed to selected respondents through Google Forms in October 2021. The Climate CAMPAIGNers project partners reviewed, pre-tested, and refined the survey before its distribution to experts from LCs.

## 3 Literature review

The adverse impacts of climate change on people's lives have been reflected in the increased number of studies in the literature on climate-friendly lifestyles and behavior changes (Mills and Schleich, 2012; Von Borgstede et al., 2013; Creutzig et al., 2018; Umit et al., 2019; Niamir et al., 2020). These studies in the literature concerning policy and individual lifestyle actions that decrease GHG emissions for climate mitigation have used both quantitative and qualitative methodological approaches (Bassett and Shandas, 2010; Geneletti and Zardo, 2016; Eisenack and Roggero, 2022; Kilkis, 2022).

In addition to studies focusing on individuals' lifestyle changes, there have been numerous studies on the climate actions of local, regional, and national authorities (Rabe, 2004; Granberg and Elander, 2007; Lutsey and Sperling, 2008; Hoppe et al., 2014; Tvinnereim et al., 2017; Salvia et al., 2021). Hsu et al. (2020) demonstrated that city-level climate mitigation efforts are shaped by “plan-level,” “city-level,” and “country-level” features. Boehnke et al. (2019) found that “good practices” regarding climate mitigation in thirteen municipalities in the Netherlands are derived from the “facilitator” role of municipalities in promoting climate-friendly actions of various actors within their borders.

Cities' climate mitigation strategies are studied with a particular focus on “transport,” “waste management,” and “urban form” (Bulkeley, 2010; Erickson and Tempest, 2015; Creutzig et al., 2016; Lamb et al., 2018). For instance, Lutsey and Sperling (2008) found that many US states have sought to adopt climate mitigation policies on “residential energy usage” and “forestry sequestration.” In this sense, local governments' mitigation strategies were based on using spaces, transportation tools, dwellings, and waste management strategies (Lutsey and Sperling, 2008). Salvia et al. (2021) examined the different scales of local governments' climate mitigation plans and their carbon neutrality regarding the relevant city's structure and size, “membership of climate networks,” and regional position.

Hence, policy actions for climate mitigation are derived from the literature review, as demonstrated in Table 1.

**Table 1:** Policy actions for climate mitigation derived from the literature review.

**Theme**	**Policy actions**	**References**
Sustainable transportation	Low-emission vehicles	Cruickshank and Kendall, 2012; Chakroborty, 2017; Kiba-Janiak and Witkowski, 2019; Miltiadou et al., 2019; Watabe et al., 2019
	Sustainable urban mobility	
Waste management	Sustainable waste management	Pereira et al., 2000; Mwanza and Mbohwa, 2017; Lagman-Bautista, 2020; Lee et al., 2020; Zhang H. et al., 2022; Zhang Z. et al., 2022; Xu et al., 2023
	Reducing pollution	
	Recycling	
	Reuse	
City Planning	Degrowth in the city's climate planning	Kristiánová and Stepankova, 2015; Gorelick and Walmsley, 2020; Kutty et al., 2020; Kiba-Janiak et al., 2021; Krähmer, 2021; Siehr et al., 2022; Khmara and Kronenberg, 2023
	Administrative and organizational structures	
	Climate action plans	
	Subsidy schemes	
	Grant programs	
	Investments	
	Policy review	
	Stakeholder involvement	
	Green and blue infrastructure strategy	
Water and air quality management	Improving air quality	Borrego et al., 2006; Liu and Jensen, 2018; Herslund and Mguni, 2019; Jonek-Kowalska, 2023
	Enhancing water management strategies	
Energy-efficient technologies	Low-carbon technologies	Amado et al., 2016; Song et al., 2017; Lu et al., 2021
	Zero-carbon technologies	
	Energy-efficient technologies	
Environmental protection	Increasing the level of protection, restoration, and regulation of the natural environment and ecosystems	Yang et al., 2016; Horne et al., 2018; Nwakaire et al., 2020; Li et al., 2022
	Addressing the urban heat island effect	
	Preparedness for extreme weather events	
Energy consumption	Reducing energy consumption from conventional sources	Sirakaya et al., 2018; Debelaya and Morozova, 2020; Shu et al., 2022; Zhang H. et al., 2022; Zhang Z. et al., 2022
	Increasing renewables	
Awareness	Raising public awareness	Wang et al., 2017; Rahimi, 2020; Zust and Jost, 2022

Regarding individuals' lifestyle changes, several studies have utilized surveys to reveal participants' climate actions and perspectives regarding “transportation,” “energy transition,” “attribution of climate change,” “emission reduction,” “lifestyle/consumption,” “diet change,” “purchase decisions” and “waste management” (Barr and Gilg, 2006; De Boer et al., 2016; Tvinnereim et al., 2017; Belaïd and Joumni, 2020; Gjerstad and Flottum, 2021). For instance, Niamir et al. (2020) examined households' practices regarding their energy investments in smart energy systems, energy-saving habits, and shifting to green(er) electricity sources. They found that financial factors, as well as social and personal values, are significant elements. Furthermore, educational level and residences' structural conditions are crucial for climate-friendly lifestyle behaviors (Niamir et al., 2020).

Accordingly, lifestyle changes to support climate action are summarized in Table 2.

**Table 2:** Lifestyle changes supporting climate mitigation derived from the literature review.

**Theme**	**Lifestyle changes**	**References**
Sustainable transportation	Public transport	Li et al., 2015; Miller et al., 2016; Rajesh et al., 2019; Aguiléra and Pigalle, 2021; Turoń, 2023; Valentini et al., 2023
	Carpooling	
	Carsharing	
	Eco-driving	
	E-mobility	
	Walking	
	Cycling	
	Avoiding short flights	
	Reducing flights for business	
Waste management	Recycling and composting	Khan et al., 2005; Fu and Liu, 2017; Lee et al., 2020; Kountouris, 2022; Yadav et al., 2022; Biresselioglu et al., 2023
	Food waste reduction	
	Disposing less and reusing more	
	Recycling water	
	Reclaiming and reusing building materials	
Energy-efficient technologies	Smart meter deployment	Nair et al., 2012; Mills and Schleich, 2014; Bularca et al., 2018; Fitriaty et al., 2018; Jnat et al., 2020; Perić et al., 2022
	PV deployment	
	Switching to an energy supplier offering electricity from renewable sources	
	House insulation	
	House renovation	
	Switching to led lighting	
	Using double or triple-glazed windows	
	Efficient use of home appliances and whitegoods	
	Purchasing energy-efficient appliances and white goods	
Energy saving and sustainable consumption	Reducing heating and cooling	Simanaviciene et al., 2013; Trotta, 2018; Zhang, 2019; Shrestha et al., 2021; Xu et al., 2021
	Washing laundry and dishes at lower temperatures	
	Reducing clothing purchases	
	Reducing printing	
	Using less water in daily life	
Dietary habits	Green diet	Bryngelsson et al., 2017; Philippidis et al., 2021; Biresselioglu et al., 2023
Working environment	Teleworking	Hook et al., 2020; O'Brien and Aliabadi, 2020; Noussan and Jarre, 2021

Concerning the motivators and barriers to climate mitigation actions of cities, various studies demonstrate a positive relationship between people's income level and climate-friendly energy investment decisions (Sardianou and Genoudi, 2013; Ameli and Brandt, 2015; Umit et al., 2019). Furthermore, local and national governments' incentives are found to be significant motivations for lifestyle changes (Niamir et al., 2020). Building characteristics of houses, household members' socioeconomic features, “environmental concerns,” and willingness for energy conservation and “waste management” are revealed as crucial reasons for individuals' climate actions (Belaïd and Joumni, 2020).

Even though municipalities have a significant role in increasing the motivation of citizens regarding climate-friendly and sustainable actions (Glaas et al., 2020), there are limits to their capabilities and impacts. Hence, it is argued that municipalities should cooperate with the business world more ambitiously and systematically (Neij and Heiskanen, 2021) and improve their climate actions with technological developments (Lassiter and Leonard, 2022).

The motivators concerning climate actions are demonstrated in Table 3.

**Table 3:** Lifestyle change motivators derived from the literature review.

**Lifestyle change motivators**	**References**
Information and education	Alexandru and Jitaru, 2007; Fischer, 2008; Bertoldi et al., 2013a,b; Shen et al., 2021; Perret et al., 2022
Goal setting and feedback	
Persuasion, incentives	
Modeling and exemplifying	
Enablement	
Encouraging	
Engagement	
Coercion	Rosenow, 2012; Bertoldi et al., 2013a,b; Moser, 2013
Restriction	

Another line of researchers has considered the essential role of cities in climate mitigation, emphasizing the fact that they can encounter difficulties due to the problematical division of responsibility among local, national, and international authorities, financial reasons, their (in)ability to manage, and lack of certainties in institutional structure (Bulkeley and Betsill, 2005; Monni and Raes, 2008; Sharp et al., 2011; Moss et al., 2015; Webb et al., 2016; Harker et al., 2017; Neij and Heiskanen, 2021). Moreover, Rickards et al. (2014) found that senior decision-makers must deal with their “local” occupational conditions and short-term circumstances, such as prestige, ties with rivals, and economic status, which can hinder their climate mitigation activities. In this regard, it is found that municipalities' climate actions reflect an intention-behavior gap since climate strategies are likely to be unchanged in various cities (Bulkeley, 2015; Van der Heijden, 2019).

The barriers concerning climate actions, as derived from the literature review, are shown in Table 4. The identified motivators and barriers are utilized in the survey design.

**Table 4:** Identified motivators and barriers utilized in the survey design.

**Type of barrier**	**Lifestyle change barrier**	**References**
Internal barriers	Difficulty with changing existing habits	Throne-Holst et al., 2008; Zhu and Geng, 2013; Le-Anh et al., 2023; López-Cózar-Navarro et al., 2023
	Personal unwillingness to change	
	Unwillingness to move from rural areas to urban ones	
	Unwillingness to move to smaller homes	
	Unwillingness to build a new and more sustainable home	
	Pessimism about the future	
	Insufficient knowledge to overcome mitigation inaction	
	Too much information to make meaningful decisions	
	Time needed to adapt to changes	
External barriers	High perceived cost of climate-beneficial actions and carbon-neutral actions	Throne-Holst et al., 2008; Wang et al., 2008; Al-Hinti and Al-Sallami, 2017; Kazemi and Kazemi, 2022
	Cost of required investments for energy efficiency upgrades	

## 4 Analysis of results

The survey is designed in two parts. The first part involves questions concerning LCs' climate change mitigation strategies, and the second part seeks to assess LCs' efforts toward lifestyle changes from the perspective of each expert.

### 4.1 Policy tools for climate mitigation

When asked to select the top three climate mitigation policies and tools their LCs prioritize, 48 of the 70 participants identified “education and enabling” as one of the top prioritized climate mitigation policies. The policy action selected by the second highest number of participants (20) as one of the three top climate mitigation policies was “regulation.”

“ICT and digitalization” emerged as the least prioritized policy tool for their respective LCs, with only two participants (both from Vilnius) selecting digital policies among their LC's top climate mitigation strategies. None of the participants from Izmir, Dublin, or Lahti considered ICT and digitalization to be top priorities for climate mitigation. This finding is interesting as previous studies have emphasized the critical role of green technologies in contributing to climate mitigation strategies (Balogun et al., 2020).

Regarding the primary policy actions implemented in the last 5–10 years and are currently being implemented and/or need to be implemented in the next 5–10 years to deal with climate mitigation in the respective relevant cities, the responses of the experts revealed that “reducing pollution” was the most favored policy action across all 11 LCs during the past 5–10 years, with a 42% preference rate.

For the timeline of the last 5–10 years, the policy actions “raising public awareness” (40%), “facilitating more sustainable waste management” (34%), “improving air quality” (33%), and “reviewing and updating of existing local policies, regulations, and guidelines” (32%) were other popular responses among participants concerning climate mitigation strategies.

Regarding current policies, participants identified “public awareness” as the most prioritized policy action/tool, with a response rate of 63 %. Looking ahead to the next 5–10 years, 61% of experts stated that their LCs would prioritize “installing low and zero carbon and energy-efficient technologies.”

Table 5 summarizes the perspectives on the highest-priority and lowest-priority policy actions for climate mitigation in the LCs.

**Table 5:** Policy actions prioritized by the highest and the lowest number of experts.

**Time frame**	**Policy actions for climate action prioritized by the highest number of LCs**	**Policy actions for climate action prioritized by the lowest number of LCs**
Past	Reducing pollution	Incorporating degrowth in the LC's climate planning
	Raising public awareness	Addressing the urban heat island effect
	Improving air quality	Increasing preparedness for extreme weather events
	Facilitating more sustainable waste management	Developing green and blue infrastructure strategy
	Reviewing and updating existing local policies, regulations, and guidelines	Encouraging reuse of materials
Current	Raising public awareness	Incorporating degrowth in the LC's climate planning
	Reducing energy consumption from conventional sources	Addressing the urban heat island effect
	Engaging key internal and external partners and stakeholders	Developing new subsidy schemes, grant programs, and investments
	Increasing recycling rates	Increasing preparedness for extreme weather events
	Developing more sustainable mobility	Installing low and zero-carbon and energy-efficient technologies
Future	Installing low and zero-carbon and energy-efficient technologies	Reducing pollution
	Reducing energy consumption from conventional sources	Developing more sustainable mobility
	Developing new subsidy schemes, grant programs, and investments	Facilitating more sustainable waste management
	Encouraging reuse of materials	Engaging key internal and external partners and stakeholders
	Increasing preparedness for extreme weather events	Incorporating degrowth in the LC's climate planning

### 4.2 Lifestyle changes for climate mitigation

Regarding the LCs' efforts toward lifestyle changes, “sustainable transportation” was selected as the top lifestyle change for climate policies that the experts find essential for their LCs. Sustainable transportation involves changing habits, such as decreasing car use and increasing energy-efficient vehicles (Steg and Gifford, 2005). Energy efficiency was the second lifestyle change selected by the experts as the most vital to address for their LCs.

Concerning the lifestyle choices that were discussed in the last 5–10 years and are currently being discussed and/or need to be discussed in the next 5–10 years to improve the LCs' climate policies, most experts (59%) opted for “waste” as the most important theme for lifestyle choices for the past 5–10 years in their LCs.

For the current era, “switching to electric cars and vehicles” is the most cited current issue in 11 LCs, with around 75% of respondents selecting it as a top priority. “Switching to electric cars and vehicles” was also the lifestyle choice to be prioritized in the coming 5–10 years by the second highest share (41%) of respondents for their LCs. Concerning the future outlook, the highest share of respondents (51%) selected “reclaiming and reusing building material” among the top priorities as the lifestyle choices for climate mitigation in their LCs.

The following Table 6, reflects the results from the survey concerning the top priorities in terms of lifestyle choices for climate mitigation in the LCs of the respondents.

**Table 6:** Lifestyle changes prioritized by the highest and the lowest number of experts.

**Time frame**	**Lifestyle changes prioritized by the highest number of LCs**	**Lifestyle changes prioritized by the lowest number of LCs**
Past	Paper waste recycling	Reducing clothing purchases
	Plastic, metal, and glass waste recycling	Reducing business flights
	Public transportation	Avoiding short flights
	Switching to LED lighting	Teleworking
	Replacing windows with double or triple-glazed versions	Green diet
Current	Switching to electric trucks	Washing laundry and dishes at a lower temperature
	Using electric vehicles	Avoiding short flights
	Teleworking	Reducing business flights
	Investing in solar panels	Green diet
	Public transportation	Smart meter deployment
Future	Reclaiming and reusing building materials	Paper waste recycling
	Using electric vehicles	Switching to LED lighting
	Recycling water	Teleworking
	Eco-driving	Reduced printing
	Renovating to low-energy and smart houses	Plastic, metal, and glass waste recycling

### 4.3 Barriers and motivators for lifestyle changes

The experts' perspectives on the lifestyle change motivators and barriers in climate policymaking highlighted the following results.

The motivator selected with a top prominence by the highest share of experts (89%) was “information and education” (89%), followed by “encouragement” (48%) and “incentives” (48%). Three experts (one each from Baku, Vilnius, and Linz) noted that their LCs did not utilize motivators for supporting climate policymaking in their respective LCs. This correlates with previous studies suggesting local or national authorities' unwillingness to boost environmentally friendly behaviors due to their political interests (Lorenzoni et al., 2007).

Although “incentives” were regarded as one of the most significant lifestyle motivators among participants, no experts from Trujillo or Cape Town regarded “incentives” among the top lifestyle motivators for supporting climate policies. There is also a distinction between the perspectives of EU and non-EU experts regarding their perspectives on “incentives.” In this sense, while 60% of the EU experts identified “incentives” as a top lifestyle change motivator, only 30% of the non-EU experts selected “incentives” as a lifestyle change motivator.

Kent (2009) highlights that policymakers are responsible for removing barriers to lifestyle changes and encouraging city residents to change their habits. The top two lifestyle changes identified by the experts concerning climate policies pertain to changing habits, emphasizing the significance of policy action in this respect. Accordingly, concerning the barriers against lifestyle changes, the highest share of participants (70%) selected “difficulty with changing existing habits” (70%) among the most significant barriers. This was followed by “unwillingness to give up personal cars” (65%), and “cost of energy efficiency upgrades” (62%) as the most significant barriers for lifestyle changes. The barrier of “unwillingness to give up personal cars” was selected by a higher share of EU countries' (72%) respondents as a highly important barrier compared to non-EU countries' experts. This result suggests that residents of EU countries have stronger ties and higher dependence on their cars as part of their lifestyles.

## 5 Discussion of survey results and alignment with the findings from the literature review

The findings demonstrate the perspectives of experts from the 11 LCs, who have professional knowledge about climate mitigation strategies, lifestyle changes, and related experience in their respective cities. Hence, the expert survey provides significant information on the climate mitigation policies, strategies and lifestyle changes, the motivators and barriers and how the LCs prioritize them. The differences between the respondents from EU and non-EU LCs also point out noteworthy findings.

In line with the existing literature, the survey findings show that, for all LCs, public awareness of climate mitigation policies is selected by the highest share of experts (63%) as a top priority strategy for policy action. Concerning the EU perspective, this result is relevant to the previous studies that emphasize the significant role of “public education” and “outreach” in climate action strategies of European countries (Grafakos et al., 2020) and for future zero-carbon technologies (Asilsoy and Oktay, 2018).

The lowest number of experts selected the policy option of “incorporating degrowth in city's climate planning” as a top priority action item for climate mitigation policies in all timelines, the LCs' past, current, and future strategies. This outcome is unsurprising given the difficulty of degrowth policy at the macro level, as it requires a deep-rooted bottom-up and top-down transformation (Deriu, 2012; Alexander and Yacoumis, 2018; Büchs and Koch, 2019).

Regarding lifestyle changes in the LCs, the popularity of “recycling” as a priority action by the respondents from all LCs, with higher shares from European LCs, aligns with the findings from the existing literature on this topic. It has been demonstrated that recycling has become one of the essential strategies in Europe through “EU directives, fiscal measures […], pricing structures, and local authority provisions” in the recent three decades (Thomas and Sharp, 2013, p.12; Yu et al., 2019).

The survey results demonstrate that “switching to electric vehicles” is selected as a top motivator for lifestyle changes toward supporting climate policies. The literature also emphasizes that switching to electric vehicles can contribute to climate policies by reducing dependence on fossil fuels, and electric vehicles “have the potential to improve the efficiency, affordability, and sustainability of the transport system” (Ortar and Ryghaug, 2019). Hence, reducing the barriers to the deployment of electric vehicles is a significant policy agenda item for policymakers (Biresselioglu et al., 2018). The expert survey also indicates that debates on switching to electric cars are more prevalent among European LCs. This is apprehensible as the European Commission has adopted several targets for sustainable transport to achieve net zero by 2050 (Statharas et al., 2019). It is also consistent with the dramatic growth of the electric car market, which achieved sales surpassing 10 million in 2022 [International Energy Agency (IEA), 2023]. In addition, the discussions on switching to electric vehicles will likely gain momentum in the non-EU LCs as more non-EU countries are willing to decrease taxes on electric vehicles. These findings are mainly related to previous studies suggesting that lifestyle changes primarily comprise transportation transformation (Gjerstad and Flottum, 2021).

A remarkably higher percentage of experts from EU countries (28%) regard “regulations” to be among their top policy tools concerning climate mitigation as compared to experts from developing countries (13%). Similarly, ~59% of the experts from EU countries stated that their respective LCs prioritize “education and enabling” as one of the top policy strategies, compared to 31% of the experts from non-EU countries.

Concerning the recent (last 5–10 years) climate mitigation strategies, experts from EU countries point to a considerably higher share of prioritization of “increasing preparedness for extreme weather events” for their LCs, as compared to experts from non-EU LCs.

The lifestyle change “avoiding short flights” provides another example of differentiation between European and non-European LCs. Accordingly, while a more significant proportion of experts from non-European LCs (48%) prioritize “avoiding short flights,” only 18% of the experts from European LCs consider it. Given the density of short flights within Europe, this finding suggests that experts from European LCs regard short flights as a vital part of the lifestyles in their cities. Similarly, the lifestyle change concerning switching to “teleworking” is remarkably more predominant among European LCs (60%) than their non-EU counterparts (26%). This pertains to the cultural differences and impacts of COVID-19, whereby remote working has become more internalized within the climate strategies of the respective European countries compared to non-EU LCs.

The expert survey also points out the significance of cultural constructs and habits in climate change mitigation and the difference between EU and non-EU LC perspectives. The “difficulty with changing existing habits” is a more prevalent barrier to lifestyle changes among European LCs (72%) than non-European LCs (63%). This suggests that city dwellers in developed countries are more attached to their daily routines and well-established habits than their counterparts in developing non-EU countries. However, this barrier is a problem regarding LCs' climate mitigation strategies that local policymakers need to address. On the other hand, unlike European LCs, “lack of technology” and “lack of authority” emerge as crucial obstacles to lifestyle changes among the non-EU LCs.

The geographical locations of the LCs also affect the prioritization of policy actions and lifestyle choices, as evidenced by the expert survey. For instance, respondents from southern LCs with a higher level of urbanization report that their cities have considered the urban heat island effect as a priority item in the policy agenda for climate mitigation in the past (last 5–10 years) and are continuing to keep it in their current agenda.

## 6 Conclusion

As cities worldwide have increased their efforts to reduce their GHG intensity, it has become more critical to gain insight into their climate mitigation strategies and what they do about their residents' lifestyle changes. In this sense, this study examined the LCs from the perspectives of locals with prior knowledge and climate action experience in their respective cities. It primarily relies on the expert survey among 89 experts across 11 LCs and a comprehensive state-of-the-art literature review.

Several factors, processes, and variables can promote environmentally friendly lifestyles and climate mitigation strategies. As actors responsible for more than 70% of CO_2_ emissions in the world, cities are crucial players in climate mitigation policies and promoting environmentally friendly lifestyles of their residents. Furthermore, this study suggests that cities stand out as significant actors, as their activities involve multiple levels (e.g., “individual,” “household,” “community”), fields (e.g., “transportation,” “recycling,” “energy,” “food,” “water” etc.) and domains of influence (e.g., social, psychological, economic and cultural).

The expert survey and the literature review demonstrate that climate mitigation strategies regarding “increasing awareness and information provision” emerge as a critical issue for the 11 LCs under examination. In this sense, context-specific, structured, and long-term policies about this topic must be considered in the LCs to generate further support for climate actions among city residents. Another significant topic, “sustainable mobility,” is highlighted by the experts' responses and the literature review. Local governments are essential in developing their infrastructure, increasing their public network, and investing in sustainable transportation. Energy efficiency is another critical policy lever for the LCs in tackling climate change, which requires local policy experts to encourage their communities to change their daily habits.

The feasibility of the LCs' climate mitigation strategies primarily relies on public acceptance and lifestyle changes among city residents. Hence, any local authority should consider motivators and barriers to climate-friendly lifestyles. In this regard, “information and education,” “encouragement,” and local authorities' “modeling and exemplifying” for lifestyle changes are significant motivators that the LCs need to address. On the other hand, the barriers to lifestyle changes that obstruct the climate actions of LCs are “difficulty with changing existing habits,” “unwillingness to give up personal cars,” and “cost of energy efficiency upgrades.” This study suggests that these barriers differ according to the income level of the countries in which the cities are located. Hence, considering their specific circumstances, the local authorities of the LCs need to adopt interactive policy formulations combining bottom-up and top-down approaches to tackle these barriers.

Overall, this study's findings contribute to potential policy formulations of cities to address climate change through climate mitigation strategies and the promotion of lifestyle changes. All cities, regardless of income level, geographical location, or the EU membership status of their countries, need to take responsibility for their respective GHG emissions and undertake climate-friendly actions through climate mitigation policies and encouraging lifestyle changes in their cities. Future studies might testify to this necessity and address the limitations of this study by expanding the survey sample to different stakeholders rather than being confined to experts' perspectives and cities worldwide other than the Lighthouse Cities.

## Data availability statement

Data concerning this article is available upon reasonable request from the corresponding author.

## Ethics statement

The studies involving humans were approved by Izmir University of Economics' Ethics Committee on Social Sciences. The studies were conducted in accordance with the local legislation and institutional requirements. The participants provided their written informed consent to participate in this study.

## Author contributions

MB: Conceptualization, Investigation, Methodology, Project administration, Resources, Supervision, Writing—original draft. ZS: Investigation, Validation, Visualization, Writing—original draft. MD: Conceptualization, Investigation, Resources, Supervision, Validation, Writing—original draft. CK-C: Formal analysis, Investigation, Methodology, Validation, Writing—original draft.
